# Differential Protective Effects of Exenatide, an Agonist of GLP-1 Receptor and Piragliatin, a Glucokinase Activator in Beta Cell Response to Streptozotocin-Induced and Endoplasmic Reticulum Stresses

**DOI:** 10.1371/journal.pone.0073340

**Published:** 2013-09-19

**Authors:** Mi-Kyung Kim, Jin-Hwan Cho, Jae-Jin Lee, Ye-Hwang Cheong, Moon-Ho Son, Kong-Joo Lee

**Affiliations:** 1 College of Pharmacy and Graduate School of Pharmaceutical Sciences, Ewha Womans University, Seoul, Republic of Korea; 2 Dong-A ST Research Institute, Yongin-si, Gyeonggi-do, Republic of Korea; University of Lancaster, United Kingdom

## Abstract

**Background:**

Agonists of glucagon-like peptide-1 receptor (GLP-1R) and glucokinase activators (GKA) act as antidiabetic agents by their ability protect beta cells, and stimulate insulin secretion. Oxidative and endoplasmic reticulum (ER) stresses aggravate type 2 diabetes by causing beta cell loss. It was shown that GLP-1R agonists protect beta cells from oxidative and ER stresses. On the other hand, little is known regarding how GKAs protect beta cells. We hypothesized that GKAs protect beta cells by mechanisms distinct from those underlying GLP-1R agonist and tested our hypothesis by comparing the molecular effects of exenatide, a GLP-1R agonist, and piragliatin, a GKA, on INS-1 cells under oxidative and ER-induced stresses.

**Methods:**

Beta cells were treated with streptozotocin (STZ) to induce oxidative stress and with palmitate or thapsigargin (Tg) to induce ER stress respectively, and the effects of exenatide and piragliatin on these cells were investigated by: a) characterizing the kinases involved employing specific kinase inhibitors, and b) by identifying the differentially regulated proteins in response to stresses with proteomic analysis.

**Results:**

Exenatide protected INS-1 cells from both ER and STZ-induced death. In contrast, piragliatin rescued the cells only from STZ-induced stress. Akt activation by exenatide appeared to contribute to its protective effects of beta cells while enhanced glucose utilization was the contributing factor in the case of piragliatin. Also, exenatide, not piragliatin, blocked changes in proteins 14-3-3β, ε and θ, and preserved the 14-3-3θ levels under the ER stress. Isoform-specific modifications of 14-3-3, and the reduction of 14-3-3θ, commonly associated with beta cell death were assessed.

**Conclusions:**

Exenatide and piragliatin exert distinct effects on beta cell survival and thus on type 2 diabetes. This study which confirmed our hypothesis is also the first to observe specific modulation of 14-3-3 isoform in stress-induced beta cell death associated with progressive deterioration of type 2 diabetes.

## Introduction

Oxidative and endoplasmic reticulum (ER) stresses, lead to beta cell loss, increased pancreatic dysfunction [1]–[4] and worsening type 2 diabetes. Oxidative stress causes beta cell death [5], while ER stress mediates beta cell dysfunction [6]. In the insulin-resistant state, accumulation of misfolded proteins also activate apoptotic pathway via ER stress [7]. Evidence that ER stress is a crucial factor in type 2 diabetes is accumulating [4], [8], [9].

Glucose-lowering agents which also prevent apoptotic loss of beta cells, or stimulate their proliferation, arrest the progression of type 2 diabetes. Exenatide, an agonist of glucagon-like peptide 1 receptor (GLP-1R), is an incretin mimetic that efficiently lowers blood glucose levels by stimulating insulin secretion [10], simultaneously preserving beta cell mass by proliferative and anti-apoptotic effects [11], [12]. The 14-3-3 protein existing in seven isoforms (β, ε, γ, η, θ, σ, and ζ), inhibits apoptosis by interfering with proapoptotic proteins [13]–[15], and regulating ER-associated stress [16]–[19]. Activation of GLP-1R in beta cells also recruits 14-3-3 protein to phosphorylated BAD, to inhibit apoptosis [20], [21]. Thus it appears that protein 14-3-3, normally present in pancreatic beta cells, plays a role in type 2 diabetes. However, which particular isoform of 14-3-3 protein is involved and/or, if it undergoes any specific changes in the process, is not known.

Glucokinase mediates glycolysis in beta cells and hepatocytes. Glucokinase activators (GKAs), small chemicals binding to glucokinase at an allosteric site, induce insulin secretion by stimulating glycolysis through conversion of glucose to glucose-6-phosphate. Under oxidative stress, GKAs stimulate the proliferation of beta cells and preserve their viability both *in vitro* and *in vivo* [22]–[24]. Previous studies on the beneficial effects of GKA on the beta cells, focused on oxidative stress-induced death.

While it has been shown that GLP-1R agonists protect cell death and promote beta cell proliferation during oxidative and ER stresses, little is known regarding how GKAs protect beta cells, other than that they do so only oxidative stress. We hypothesized that the mechanisms by which GLP-1R agonists and GKAs protect beta cells are distinct and tested this hypothesis by comparing the molecular events characterizing the effects of exenatide, a GLP-1R agonist, and piragliatin, a GKA, on INS-1 cells under oxidative and ER-induced stresses. We treated beta cells with streptozotocin (STZ) to induce oxidative stress and with palmitate or thapsigargin (Tg) to induce ER stress respectively, and investigated the effects of exenatide and piragliatin on these cells. We found that exenatide completely rescued beta cells from both stresses, while piragliatin prevented beta cell death induced by STZ, but not by palmitate or Tg. We also found, by proteomic analyses proteomic analyses combining 2D-PAGE with nanoUPLC-ESI-q-TOF tandem mass spectrometry that modified isoforms of protein 14-3-3 differentially appeared in cells treated with exenatide or piragliatin under oxidative and ER stresses and confirmed that protein14-3-3θ plays a prominent role in beta cell survival.

## Materials and Methods

### Materials

Exenatide was obtained from American Peptide (Sunnyvale, CA). Piragliatin, discontinued in a phase II development [25] was synthesized in Dong-A (Yongin, Korea). P38 kinase inhibitor, SB203580 and PI3 kinase inhibitor, LY294002 were obtained from Calbiochem (La Jolla, CA, USA). JNK inhibitor, SP600125 and all other chemicals, unless otherwise specified, were obtained from Sigma (Saint Louis, MO, USA). All cell culture reagents were from Invitrogen (Carlsbad, CA, USA). Isoform specific anti-14-3-3-protein antibodies, anti-phosphoJNK/JNK antibodies were from Santa Cruz Biotechnology (Santa Cruz, CA, USA). Anti-phosphoAkt/Akt antibodies were purchased from Cell Signaling Technology (Beverly, MA, USA). Goat anti-mouse and anti-rabbit antibodies conjugated with horse radish peroxidase were from Bio-Rad Laboratories (Hercules, CA, USA) and Cell Signaling Technology (Beverly, MA, USA), respectively.

### Cell culture

Rat insulinoma cell line, INS-1, was kindly provided by Prof. Kang of Ajou University, Suwon, Korea [26]. INS-1 cells were maintained in RPMI 1640 (11 mM glucose), supplemented with 10% fetal bovine serum, 1 mM sodium pyruvate, and 10 mM HEPES, at 37°C in a humidified atmosphere (5% CO_2_). All experiments were performed with INS-1 cells between the 15^th^ and 23^rd^ passages. INS-1 cells were plated (7×10^4^ per well in a 96-well plate) at 48 h before overnight serum-deprivation in RPMI media with 5.6 mM glucose and treated with each drug for the indicated time at 0.2% final dimethylsulfoxide (DMSO) concentration.

### Beta cell viability

We used three reagents to induce beta cell death, STZ, palmitic acid and Tg. For STZ-induced beta cell death, serum-starved cells were pretreated with indicated concentrations of exenatide or piragliatin for 1 h, and then treated with 2 mM STZ dissolved in 0.1 M citrate buffer (pH 4.5), or with citrate buffer alone, in serum- and glucose-free media for 1 h. The cells were then recovered in serum-free and 5.6 mM glucose-containing media and treated again with each drug for additional 17 h. For palmitic acid-induced ER stress, serum-starved cells were cultured in RPMI1640 media supplemented with 1% fatty acid-free albumin and 5.6 mM glucose, containing 0.8 mM palmitic acid and each drug for 24 h. In the case of Tg which inhibits sarco/endoplasmic reticulum Ca^2+^-ATPase (SERCA), and causes the depletion of ER calcium stores and activation of apoptosis [27], the serum-starved cells were treated with 0.3 µM Tg and each drug for 6 h in serum-free and 5.6 mM glucose-containing media. Finally, cell viability was assessed by determining cellular ATP levels. Briefly, 100 µl of reconstituted CellTiter-Glo™ reagent (Promega, Madison, WI, USA) was added to media, the plate was agitated for 2 min and ATP was measured using luminometer using a LmaxII384 (MDC, Sunnyvale, CA, USA). Cell viability was expressed as a percentage of control. For kinase inhibitor effects, the cells were pretreated for 1 h with 10 µM of each kinase inhibitor and co-treated with each stress inducer alone, or with stress inducer combined with exenatide or piragliatin.

### MTT assay

Glucose-6-phosphate generation augments glycolytic or pentose phosphate pathways, whereby cellular NAD(P)H levels increase according to the glucose utilization. NADPH-coupled processes have direct impact on cell survival [28]. MTT colorimetric assay, which measures reduction of tetrazolium to formazan, detects intracellular NAD(P)H levels correlating with glucose oxidation and utilization [29]. This was modified and performed as described previously [30]. Briefly, half an hour after 1 h STZ treatment, 50 µl MTT solution was added and cells were incubated for additional 2 h (0.4 mg/ml MTT). After discarding supernatant, formazan formed was dissolved in 200 µl of DMSO and measured at 540 nm using a SpectraFluor (Tecan, Männedorf, Switzerland).

### Immunoblotting

INS-1 cells were harvested and lysed using RIPA buffer supplemented with EDTA-free complete protease inhibitor cocktail (Roche Diagnostics, Indianapolis, IN, USA), 1 mM sodium fluoride, 2 mM sodium ortho-vanadate and 2 mM PMSF on ice for 20 min. After centrifugation at 13,000× g for 15 min at 4°C, total protein in the lysate was determined using the bichinchonic acid kit (Pierce, Rockford, IL, USA). Ten micrograms of total protein for beta actin or fifteen micrograms for other target proteins were separated on a 4–12% precast Bis-Tris gel (Invitrogen, Carlsbad, CA, USA) in MOPS buffer and transferred to PVDF membranes. Non-specific binding was blocked with PBS containing 5% bovine serum albumin and 0.1% Tween-20. Dilutions of antibodies used in blocking solution were: Isoform specific anti-14-3-3 antibodies (1∶200), anti-phosphoJNK (^183^Thr/^185^Tyr) (1∶500), anti-JNK (1∶1,000), anti-phosphoAkt (^473^Ser) (1∶1000), anti-Akt (1∶1,000) and secondary antibodies (1∶2,500). Blots were developed with FEMTOMAX-110 reagents (Rockland Immunochemicals, Gilbertsville, PA, USA), and imaged on ChemiDoc system (Bio-Rad Laboratories, Hercules, CA, USA).

### Two-dimensional gel electrophoresis-based proteomics

Cellular proteins were extracted after lysing with a buffer containing 7 M urea, 2 M thiourea, 4% (v/v) CHAPS, 2% ampholine (1.5% pH 3–10, 0.5% pH 5–7) and 65 mM dithiothreitol (DTT), centrifuged at 20,000× g for 30 min at 25°C, and the supernatants left at room temperature for 1 h to ensure protein solubilization and denaturation. Total protein concentrations were determined using a 2-D Quant Kit (GE Healthcare, Piscataway, NJ, USA). One hundred micrograms of total protein were loaded onto the strip gels rehydrated for 12 h (18 cm, pH 4–7) with rehydration buffer (7 M urea, 2 M thiourea, 2% (v/v) CHAPS, 2% IPG buffer, pH 4–7). They were then electrofocused in a manifold cup-loading system with IPGphor (GE Healthcare, Piscataway, NJ, USA). The separated strip gels were equilibrated in a buffer containing 6 M urea, 2% SDS, 50 mM Tris-Cl, pH 8.8, 30% glycerol with 65 mM DTT for 15 min. After a second equilibration with the same buffer but containing 2.5% (v/v) iodoacetamide instead of DTT and bromophenol blue for 15 min, the strip gels were applied to 1.0 mm thick 10% acrylamide gels and sealed with 0.25% agarose. SDS-PAGE was carried out at 15 mA overnight using a PROTEAN II ×l 2-D Cell apparatus (BIO-RAD, Hercules, CA, USA). Each set of gels was silver-stained simultaneously in the same tray and scanned using an Image Scanner III (GE Healthcare, Piscataway, NJ, USA) at least in triplicate. Spot detection, matching, normalization and quantification were automatically carried out using the ProgenesisSameSpots v5.0 software (Nonlinear Dynamics, Newcastle, UK). The protein spots showing at least 1.5-fold differences in all three replicates were subjected to MS/MS analysis for protein identification.

### Protein identification by UPLC-ESI-q-TOF tandem MS

In order to identify the proteins and their modifications, the differentially expressed protein gel spots were destained and digested with trypsin and the resulting peptides were extracted as previously described [31]. Lyophilized peptide extracts were dissolved in 10% acetonitril containing 1.0% formic acid, desalted and separated using trap column cartridge (5 µm particle size, NanoEase™ dC18, Waters Co., Milford, MA, USA) with an integrated electrospray ionization SilicaTip™ (±10 µm i.d., New Objective, Woburn, MA, USA). Chromatography was performed on line to mass spectrometer (Q-tofUltima™ global, Waters Co., Milford, MA, USA). MS/MS spectra were matched against amino acid sequences in SwissProt. All reported assignments were verified by automatic and manual interpretation of spectra from Mascot and MOD^i^ (Korea, http://prix.hanyang.ac.kr/modi/; [32]) in a blind mode.

For post-translational modification analysis, peptides were analyzed by nanoAcquity™ UPLC™/ESI/MS (SYNAPT™ HDMS™, Waters Co., Milford, MA, USA). Following positive identification from database search (Mascot), all identified peptides were non-redundantly excluded in the next run analysis using SEMSA technique until almost full sequence coverage was obtained [31]. MS/MS spectra were matched against amino acid sequences in NCBI and SwissProt. All reported assignments were verified by automatic and manual interpretation of spectra from Mascot and Proteinlynx search engine and MOD^i^ in a blind mode [32], [33].

### Silencing protein 14-3-3θ

Protein 14-3-3θ was silenced in INS-1 cells by transfection with 100 nM ON-TARGET plus SMARTpool siRNAs for rat 14-3-3θ using DharmaFECT 1 transfection reagent (Dharmacon, Lafayette, CO, USA) for 48 h in OPTI-MEM I media supplemented with 5.6 mM glucose. At the end of the experiment, cellular viability was assessed using CCK-8 reagent (Dojindo Laboratories, Kumamoto, Japan) according to the manufacturer's instruction and cells were subsequently harvested for immunoblotting.

## Results

### Relative protective effects of exenatide and piragliatin on stress-induced beta cell death

**Figure 1:**
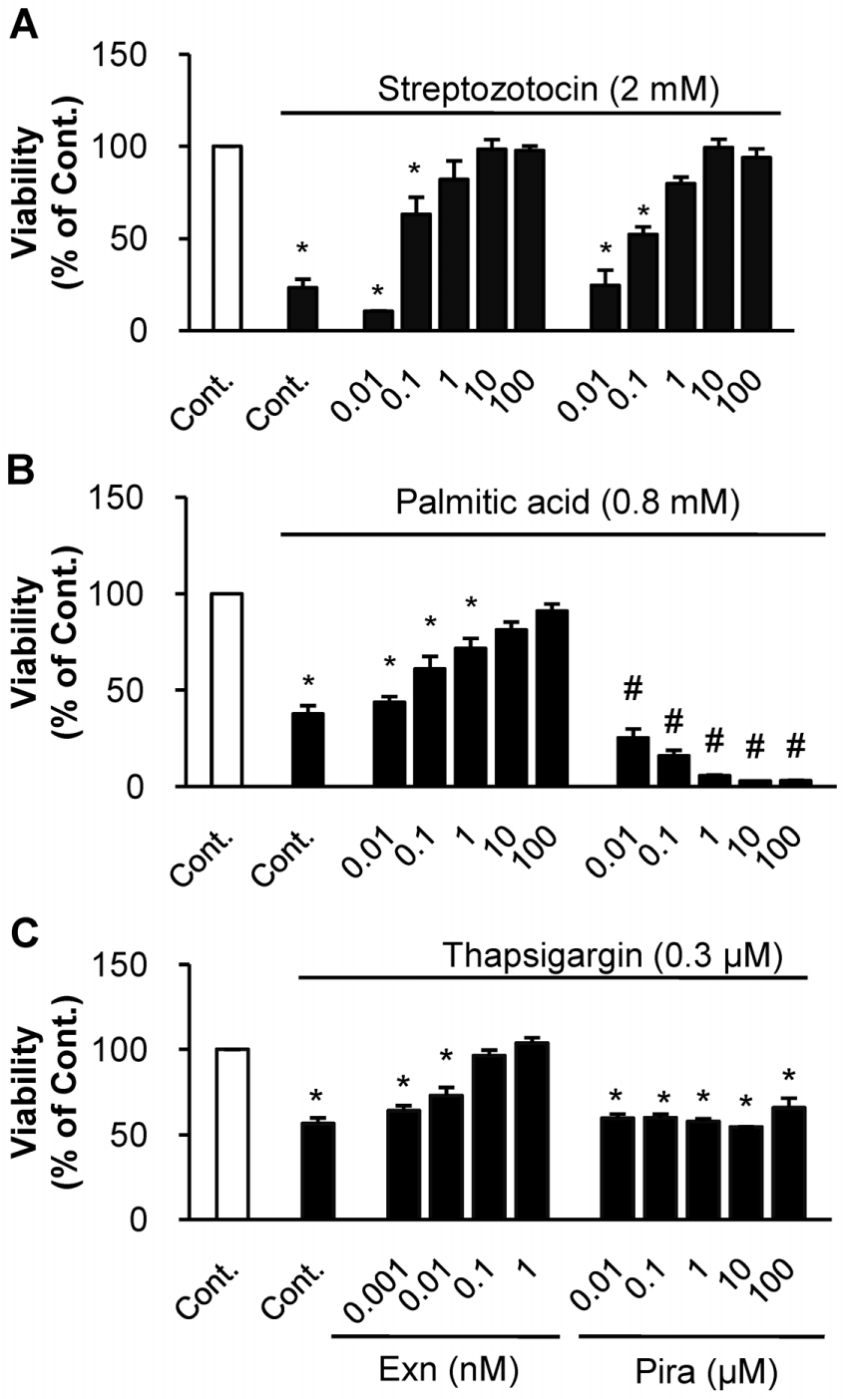
Effects of exenatide and piragliatin on beta cell death in INS-1. (A) INS-1 cells, serum-starved overnight, were pre- and post-treated with each drug with or without 2 mM STZ for 1 h. (B) INS-1 cells serum-starved overnight were treated with or without 0.8 mM palmitate for 24 h. (C) INS-1 cells serum-starved overnight were treated in the absence or presence of each drug, with or without 0.3 µM Tg for 6 h. At the end of each experiment, cell viability was assessed by cytosolic ATP levels and data from three individual experiments were presented as mean ± S.E. *, P<0.05 vs. control in the absence of each death inducer; #, both P<0.05 vs. control in the presence of palmitate by Bonferroni's post hoc analysis.

We compared the abilities of exenatide and piragliatin to protect beta cells from death induced by two stresses. Serum starved INS-1 cells were treated with 2 mM STZ for 1 h, to induce oxidative stress and recovered overnight in serum free media. After STZ treatment, we found that their viability decreased to 23.2% of control citrate buffer alone. When exenatide or piragliatin was treated during pre- and post-STZ treatments, cell viability increased in a dose-dependent manner in both cases (Fig. 1A). Maximum protective responses were obtained at doses of 10 nM exenatide and 10 µM piragliatin.

Next, we employed palmitic acid and Tg to induce ER stress-induced cell death. When starved INS-1 cells were treated with 0.8 mM palmitic acid for 24 h, their viability decreased to 37.7% (Fig. 1B). When cells were treated with palmitate and various concentrations of exenatide or piragliatin, exenatide protected the starved cells from palmitate-induced death in a dose-dependent manner. In contrast, piragliatin alone at up to 100 µM, or exenatide alone up to 0.1 µM showed no cytotoxicity for 24 h in the absence of death inducer (Fig. S1), but exacerbated the palmitate-induced cell death even at low concentrations. We did a similar comparative studies replacing palmitate, with another ER stress inducer Tg. When INS-1 cells were treated with 0.3 µM Tg for 6 h, their viability was reduced to 43.4%, while exenatide treatment completely blocked Tg-induced cell death at 0.1 nM: piragliatin could not rescue this cell death even at 100 µM (Fig. 1C). These results demonstrate that exenatide protects INS-1 cells from STZ-, palmitate- and Tg-induced cell deaths. In contrast, piragliatin can protect the cells from STZ-induced cell death, but not from ER stress-induced cell death. Based on these results, we selected the concentrations of the two drugs exerting full effect; 10 nM for exenatide and 10 µM for piragliatin for further studies.

### Contribution of piragliatin-enhanced glucose utilization

**Figure 2:**
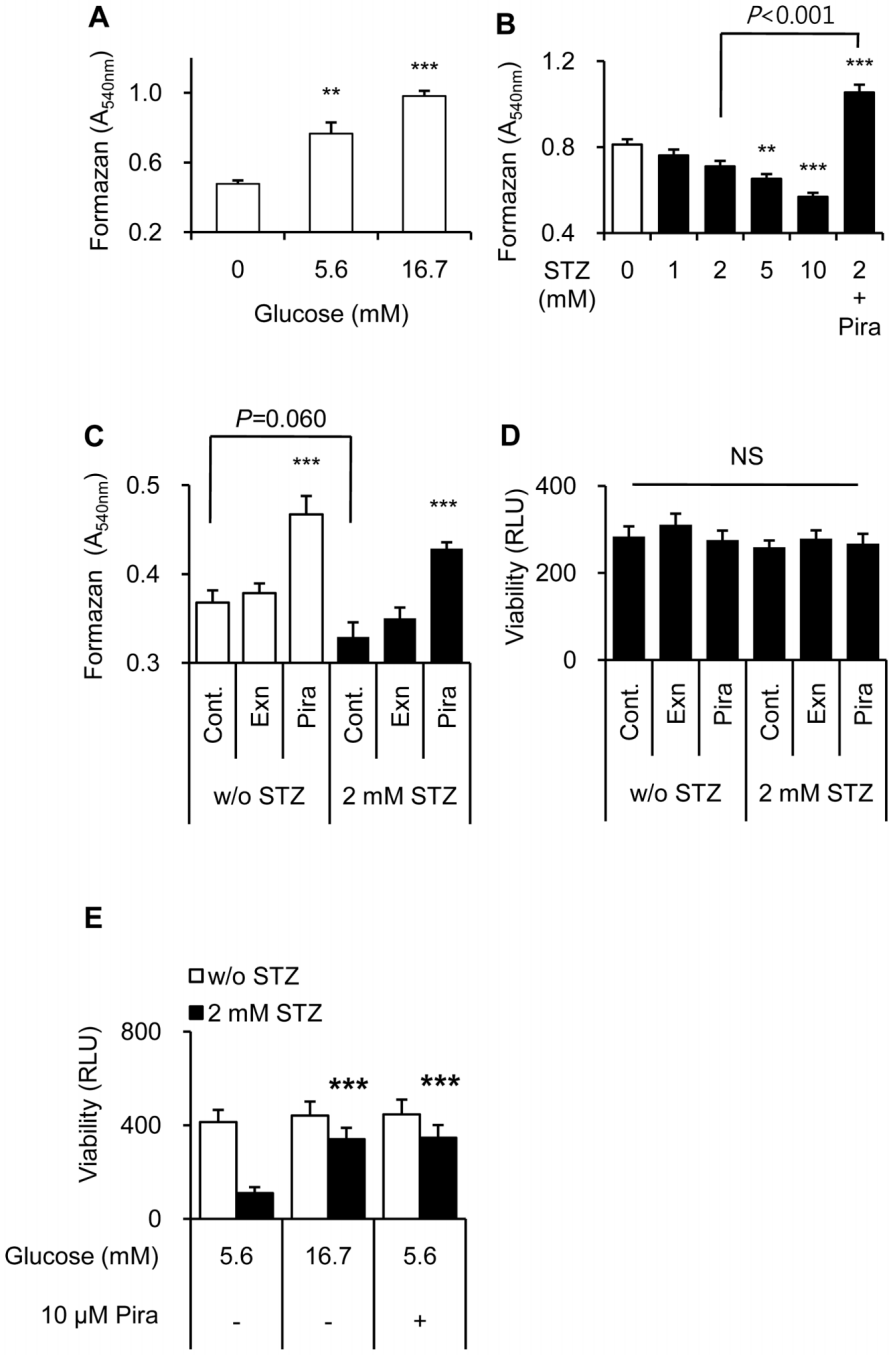
Contribution of increased glucose utilization by piragliatinin the STZ-induced beta cell death. (A–C) Intracellular NAD(P)H levels were assessed by MTT assay for 2 h starting from 0.5 h after recovery to STZ- and serum-free and 5.6 mM glucose-containing media. (D) Cellular ATP levels were measured 2.5 h after recovery at 5.6 mM glucose. (E) Eleven hours after recovery at 5.6 mM or 16.7 mM glucose in the absence (white column) or presence (black column) of 2 mM STZ, cellular ATP levels were assessed. Data from 5 to 10 determinants are presented as mean ± S.E. ** and ***, P<0.01 and P<0.001 vs. each control, respectively by Bonferroni's post hoc analysis.

To explain the distinct effect of piragliatin in STZ-induced cell death, we examined how cellular NAD(P)H levels change with glucose utilization. After 3.5 h-treatment, NAD(P)H levels rose in a glucose-dependent manner (Fig. 2A) and decreased the presence of STZ (Fig. 2B). In the early phase of treatment with piragliatin, glucose utilization significantly increased both in the absence or presence of STZ at 5.6 mM glucose (Fig. 2C), without significant differences in cell viability among treatment groups (Fig. 2D). Furthermore, increased glucose utilization at 16.7 mM glucose during 17 h-recovery period also mostly prevented STZ-induced cell death, as did piragliatin treatment at 5.6 mM glucose (Fig. 2E), underscoring the contribution of increased glucose utilization.

### Exenatide and piragliatin differently affect the stress-related kinases

**Figure 3:**
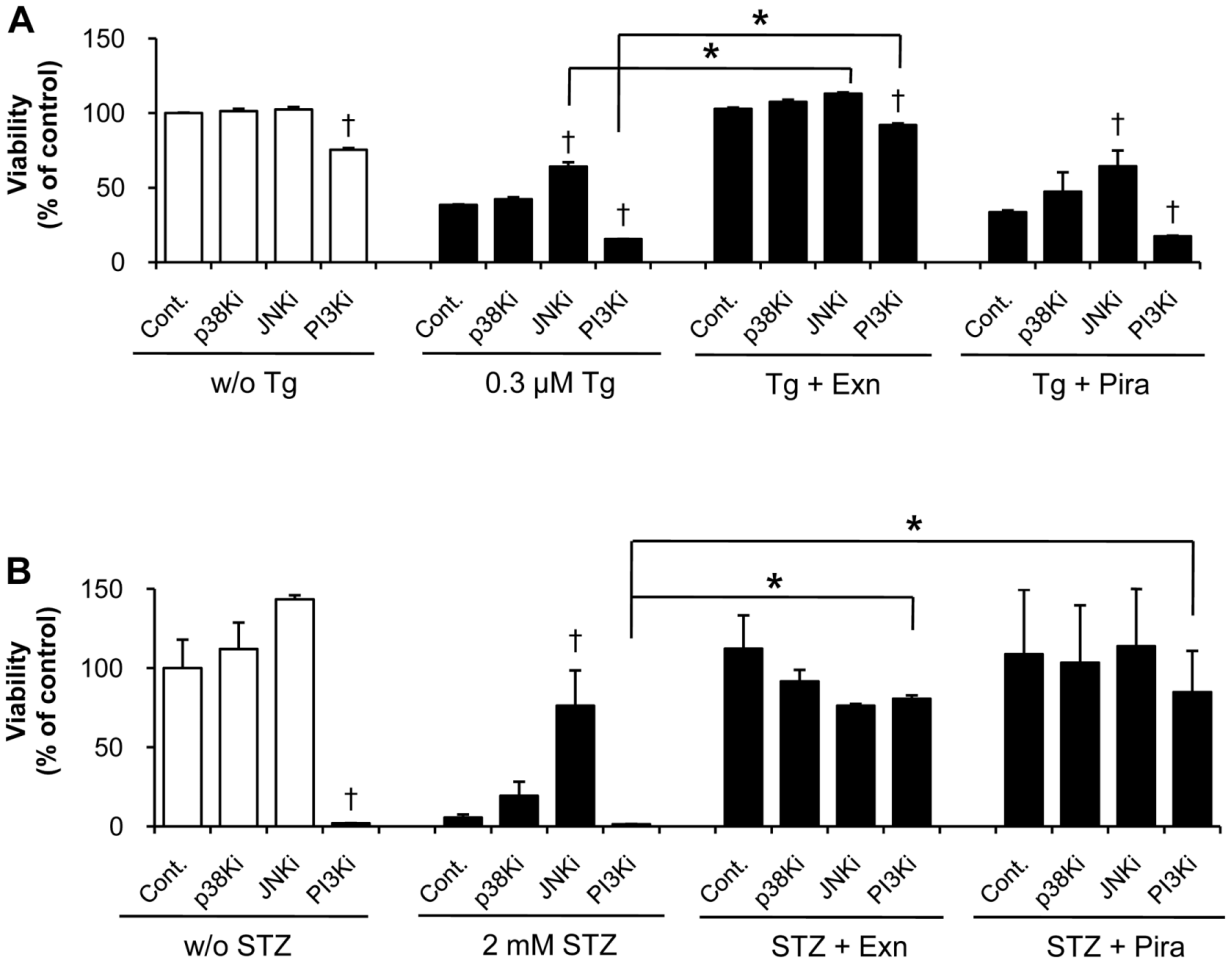
Beta cell protection by exenatide and piragliatin in the presence of various kinase inhibitors. (A) In Tg-induced cell death, INS-1 cells were pretreated with 10 µM of various kinase inhibitors for 1 h. The cells were then treated with 0.3 µM Tg and 10 µM kinase inhitor in the presence or absence of either 10 nM exenatide or 10 µM piragliatin for 6 h. (B) In STZ-induced cell death, INS-1 cells were pretreated with 10 µM of various kinase inhibitors in the presence or absence of either 10 nM exenatide or 10 µM piragliatin, and then were transiently treated with 2 mM STZ in glucose- and serum-free media for 1 h. Thereafter, cells were recovered in serum-free and 5.6 mM glucose-containing media in the presence of kinase inhibitor and each drug for 17 h. At the end of each experiment, cell viability was assessed by cytosolic ATP levels. Data from 3 to 5 individual determinants were denoted as mean ± S.E. †, P<0.05 vs. each control in a same treatment set by Bonferroni's post hoc analysis; *, P<0.05 between two treatments by Student's t-test.

To determine the modes of action of exenatide and piragliatin in preventing cell death, we examined the effects of kinase inhibitors on cell viability in response to stress inducers and/or each drug. PI3 kinase inhibitor, in the absence or presence of Tg and STZ, decreased cell viability. In contrast, JNK inhibitor significantly inhibited the stress-induced cell death without significant effects on cell viability in the absence of Tg and STZ (Fig. 3A and 3B). In contrast to inhibition of P13 kinase and JNK, inhibition of p38 kinase had no significant effects under the same conditions.

As shown in Fig. 3A, only exenatide rescued Tg-induced cell death and also significantly reversed the cell death induced by Tg and PI3 kinase inhibition. Exenatide acted similarly in the presence of JNK inhibitor (*P*<0.05). However, piragliatin showed no effect on Tg-induced cell death. With STZ-treatment, piragliatin, like exenatide, prevented cell death in the presence of PI3 kinase inhibitor (Fig. 3B). However, these effects of piragliatin and exenatide disappeared when combined with JNK inhibitor, reflecting the difference in the mode of action of exenatide upon stress condition.

**Figure 4:**
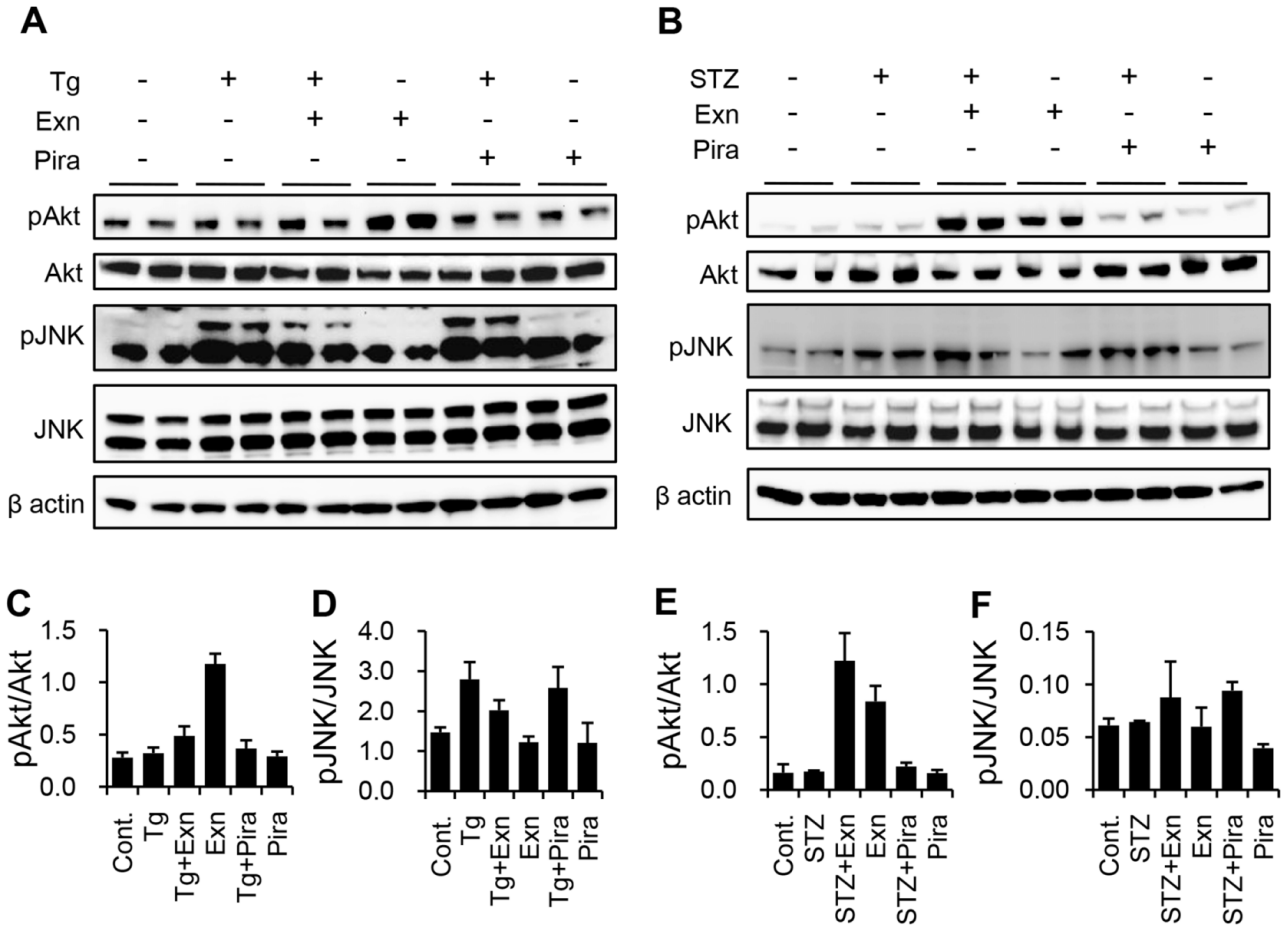
Differential effects of exenatide and piragliatin on the activation of Akt and JNK. (A, C, D) In Tg-induced cell death, INS-1 cells were harvested 1 h (for pAkt and Akt) and 3 h (for pJNK and JNK) after treating with 0.3 µM Tg in the presence or absence of either 10 nM exenatide or 10 µM piragliatin. (B, E, F) In STZ-induced cell death, INS-1 cells were pretreated with either 10 nM exenatide or 10 µM piragliatin. Following 2 mM STZ treatment for 1 h in the absence of glucose and serum, cells were harvested 0.5 h after recovery to STZ-free media with 5.6 mM glucose and each drug. In each experiment, the cell lysates were separated on 4–12% precast Bis-Tris gels, transferred onto PVDF membranes, and immunostained with phosphoAkt, Akt, phosphoJNK, and JNK antibodies. Beta actin was a representative image from JNK treatment set. (C–F) Quantified results of A and B (N = 2).

Because activation of Akt, a downstream PI3 kinase effector, can reverse the inhibition of PI3 kinase, we examined the appearance of phosphoAkt with phosphoJNK at each optimal time point from preliminary experiments. Exenatide increased Akt activation and decreased JNK activation, but piragliatin did not affect the activation of both Akt and JNK in Tg-induced cell death (Fig. 4A, 4C–D). In STZ-induced cell death, exenatide prominently increased phophoAkt levels without affecting JNK activation, while piragliatin marginally increased phosphoAkt (Fig. 4B, 4E–F). These results shown in Fig. 3 and 4 in tandem support that beta cell protection by both exenatide and piragliatin occurs via Akt activation in STZ treatment and the protective effect from exenatide via Akt activation with JNK inhibition in Tg treatment.

### Identification of the 14-3-3 isoforms involved in stress-induced beta cell death

**Figure 5:**
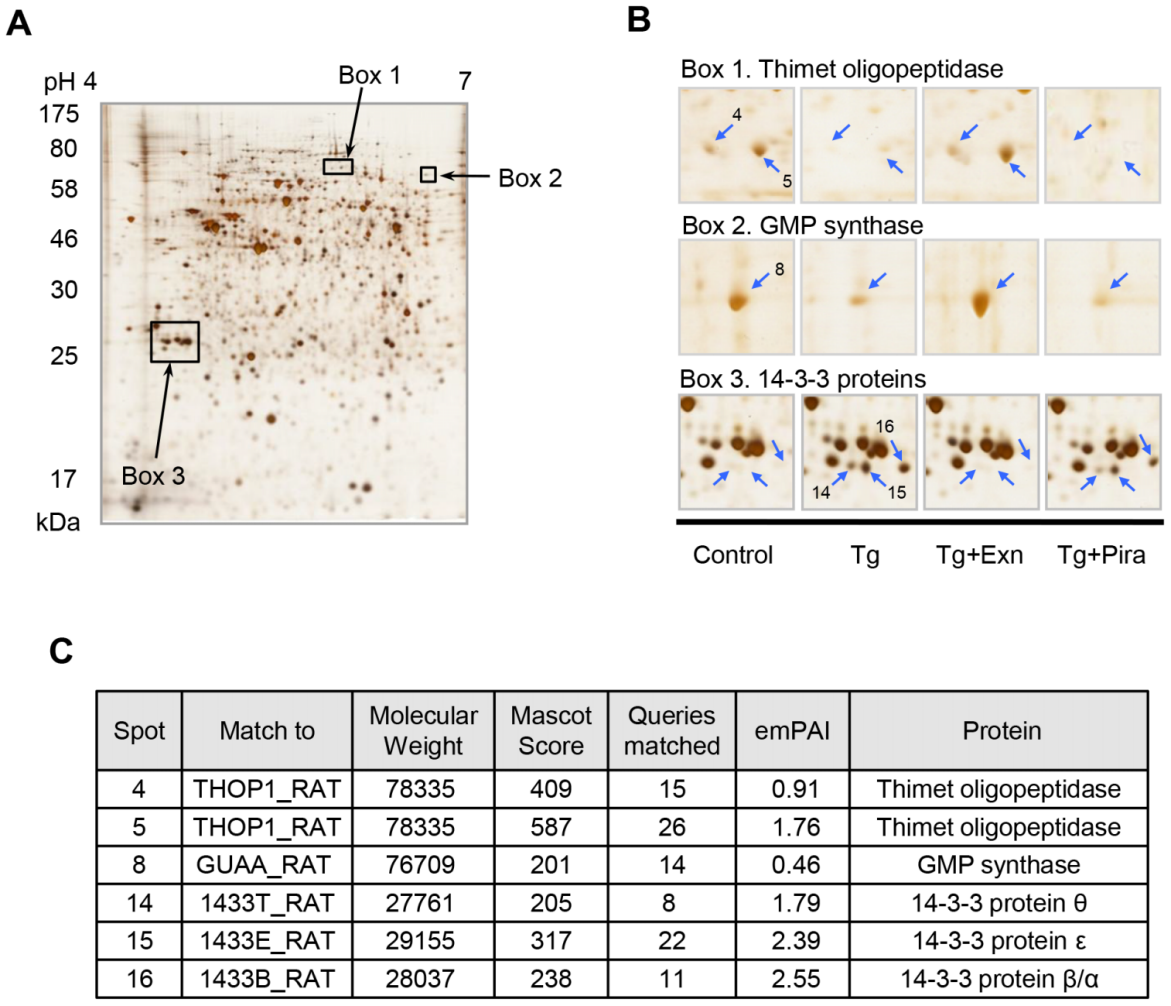
Identification of protein spots appearing in exenatide and piragliatin treated INS-1 cells by differential 2D analysis. Six hours after treatment with 0.3 µM Tg in the presence or absence of either 10 nM exenatide or 10 µM piragliatin, INS-1 cells were harvested and lysed for 2D analysis. After electrofocusing on strip gels (18 cm, pH 4–7), samples were separated on 10% acrylamide gels, silver stained, and scanned for image analysis (A). Representative images of six protein spots are shown in (B). (C) Each protein spot was cut out, destained and in-gel digested by trypsin. Digested samples were extracted, separated, and identified using nanoUPLC-ESI-q-TOF tandem MS.

**Figure 6:**
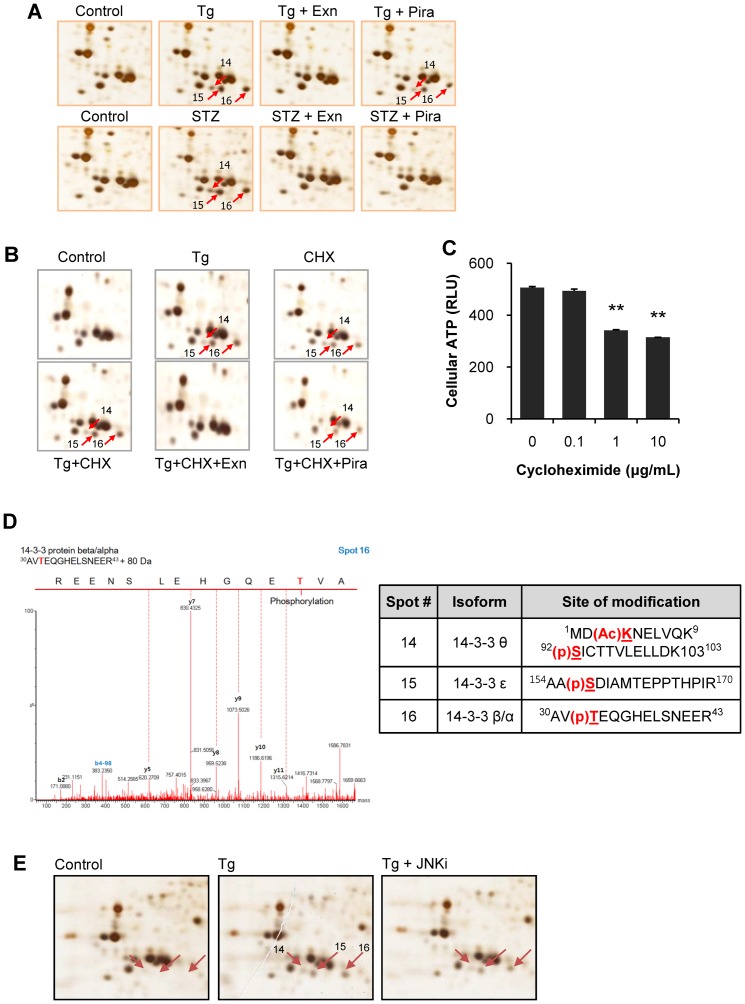
Identification of post-translationally produced potein 14-3-3 isoforms. (A) Appearances of three spots of 14-3-3 isoforms accorded with beta cell death regardless of the type of death stimulus. (B) After pretreatment of 10 µg/ml cycloheximide in INS-1 cells for 0.5 h, cells were treated with 0.3 µM Tg in the presence or absence of cylcloheximide and either 10 nM exenatide or 10 µM piragliatin. (C) In a separate experiment, cycloheximide-induced cytotoxicity was assessed by cellular ATP levels 6.5 h after cycloheximide treatment. (D) A representative image of related MS spectrum and identified modifications of 14-3-3β, ε, and θ. (E) After 20 µM JNK inhibitor was pretreated for 1 h and treated with 0.3 µM Tg, three spots of 14-3-3 proteins were determined on 2D gels.

We attempted to determine the systemic changes of INS-1 cells in response to protective effect of exenatide and piragliatin in beta cell death induced by STZ and Tg. Cell lysates were separated on 2D-PAGE and the differentially expressed proteins were identified by peptide sequencing using UPLC-ESI-q-TOF tandem MS. Fig. 5 shows six protein spots significantly altered by Tg treatment. These were identified as 14-3-3β, θ, and ε whose appearances accorded with cell death, and thimet oligopeptidase and GMP synthase with cell survival, respectively (Fig. 5B, 5C). These spot changes were reversed by exenatide treatment, but not by piragliatin. We also found that these three 14-3-3 spots also appeared in STZ-induced cell death. However, they disappeared after exenatide or piragliatin treatment, indicating that their appearance accorded with stress-induced beta cell death regardless of the type of the death stimuli (Fig. 6A).

To determine whether the appearance of the new spots was dependent of protein synthesis, we performed the same experiment in the presence of cycloheximide, a protein synthesis inhibitor. We found that the protein spots14, 15, and 16 also persisted after cycloheximide treatment, suggesting that they are produced during post-translational modification, and not during their biosynthesis (Fig. 6B). Cycloheximide alone as a control also induced three 14-3-3 spots due to the cytotoxicity (Fig. 6C). Fig. 6D shows that these spots are phophorylated forms of 14-3-3β, ε, or θ.

We next explored the contribution of JNK to the generation of these modified 14-3-3 isoforms by pre- and co-treatment of Tg with 20 µM JNK inhibitor. These treatments had little or no effect on the appearance of the three spots suggesting that the modified 14-3-3s are not the products of JNK (Fig. 6E).

### Changes in 14-3-3 isoform levels in response to Tg and STZ treatments

**Figure 7:**
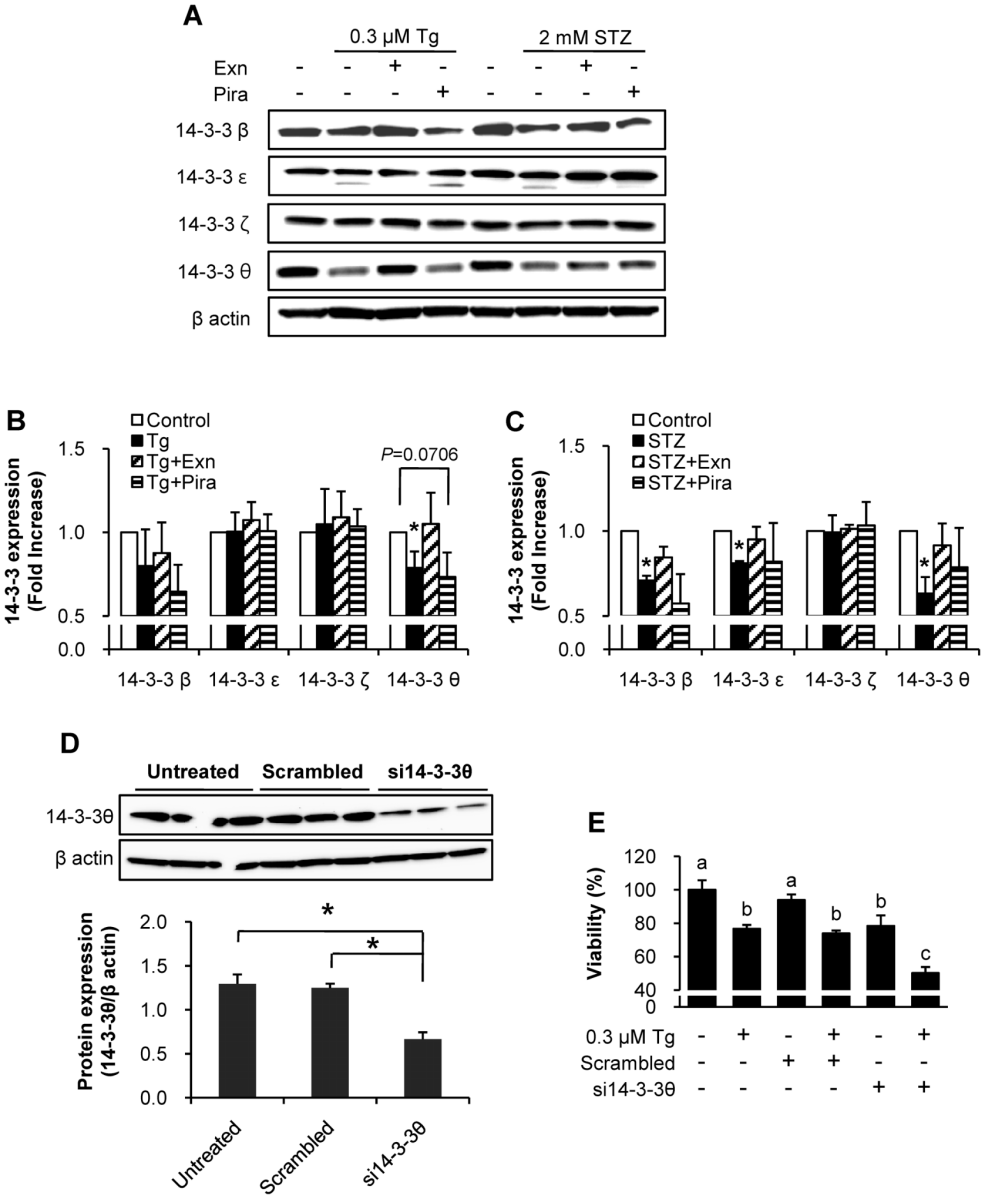
Levels of protein 14-3-3θ negatively correlate with the viability of INS-1 cells. (A–C) Levels of protein 14-3-3 isoforms were assessed by immunoblotting at the end of treatment. (A) A representative image, (B) quantification data for Tg treatment and (C) for STZ treatment denoting mean ± S.E. from three different samples. Control, Tg or STZ alone, Tg or STZ plus 10 nM exenatide, and Tg or STZ plus 10 µM piragliatin; *, P<0.05 vs. each control by Bonferroni's post hoc analysis. (D–E) INS-1 cells were transfected with specific siRNA for 14-3-3θ, control scrambed siRNA, or without siRNA for 48 h. (D) Knockdown of 14-3-3θ was confirmed by immunoblotting anti-14-3-3θ antibody. *, P<0.05 by Student's t-test. (E) At the end of treatment in a same experiment, cellular viability was assessed by adding CCK-8 reagent (Dojindo Laboratories, Kumamoto, Japan) in supernatant and incubating cells for 1 h at 37°C followed by measuring optical density at 450 nm according to the manufacturer's instruction. Different alphabet characters indicate different statistical significance (P<0.05 by Bonferroni's post hoc analysis).

We investigated whether the protein levels of 14-3-3 isoforms change. We found that the four isoforms (β, ε, ζ, and θ) were differentially expressed in response to Tg and STZ treatments (Fig. 7A). Protein levels of 14-3-3ζ did not change after both Tg and STZ treatments. Levels of 14-3-3ε significantly decreased only in STZ treated cell death but not in Tg treated death. Faint bands of 14-3-3ε which appeared after both Tg and STZ, disappeared in Tg and exenatide treatments and in STZ treated cells with either exenatide or piragliatin. Among the four isoforms, 14-3-3θ significantly decreased after both Tg- and STZ-treatments and but was restored after exenatide treatment. Piragliatin showed different effects on the levels of 14-3-3θ in Tg- and STZ-treatments, indicating that levels of 14-3-3θ alter in relation to beta cell survival under the two different stresses. Similar results were obtained with the three different sets of lysates from cells treated with Tg or STZ combining with exenatide or piragliatin (Fig. 7B and 7C). 14-3-3β and 14-3-3θ isomers changed similarly (Fig. 7B), but 14-3-3β was restored only by exenatide treatment in the presence of STZ (Fig. 7C).

### Silencing protein14-3-3θ reduced the viability of INS-1 cells

To explore the role of 14-3-3θ on beta cell death, we silenced it using specific siRNA in INS-1 cells and assessed the cell viability. When the levels of 14-3-3θ were reduced by 47.5% (Fig. 7D), INS-1 cell viabilities of untransfected and scrambled siRNA control, respectively, decreased by 21.7% and 16.6% (Fig. 7E). Moreover, Tg-induced cell death was significantly enhanced by knock-down of 14-3-3θ with reduction of cell viability of 35.9%, while 23.4% and 21.3% in untransfected and scrambled siRNA conditions, respectively (Fig. 7E). These results show that 14-3-3θ levels positively correlate with the viability of INS-1 cells.

## Discussion

This study confirms our hypothesis that the protective effects of exenatide and piragliatin in beta cell death are exerted through different mechanisms and pathways. Reduced insulin secretion and beta cell loss, are the major hallmarks of type 2 diabetes. Beta cells cannot be readily restored due to their long lifespan and low turnover [3], [6]. Thus beta cell apoptosis, but not their defective regeneration, is a key factor in the progression of type 2 diabetes. Elevated free fatty acids, alone, or in conjunction with hyperglycemia were suggested as causes of pancreatic beta cell loss and deterioration of type 2 diabetes [4], [34] among others. Therefore, we compared the beta cell protective potentials and the underlying mechanisms of two different classes of anti-diabetic agents, exenatide and piragliatin.

Exenatide completely prevented beta cell death caused from palmitate, Tg, and STZ, while piragliatin prevented only cell death induced by STZ. This reason for this difference was explored by assessing glucose utilization and stress-related kinase activation. Employing proteomic tools, the proteins differentially expressed in response to each treatment were identified, as isoforms of 14-3-3. Further studies showed that 14-3-3θ was the isoform that plays a prominent role in beta cell survival.

Under stress conditions, some kinases are known to be activated by phosphorylation, leading to survival or apoptosis [35], [36]. In this study, PI3 kinase inhibitor, after both Tg and STZ treatments, decreased basal cell viability, while JNK inhibitor only inhibited the stress-induced cell death. These results confirm previous reports of PI3 kinase inhibition-induced [37] and stress-dependent apoptosis by JNK activation [27] in beta cells.

Exenatide significantly enhanced Akt activation in STZ and Tg treatments, while it inhibited JNK activation only in Tg-induced beta cell death. Enhancement of Akt activation by exenatide was commonly observed under H_2_O_2_ induced stress [38] and human islet amyloid-induced damage [39]. However, information on the effect of exenatide on stress-induced JNK activation was contradictory [38], [39]. Our findings support that Akt activation plays a key role in the protective effect of exenatide in both types of beta cell death.

Previous studies reported that increased glucokinase activity resulted from GKA reduced beta cell death due to high glucose [40] and oxidative stress [22]–[24]. In this study, piragliatin rescued beta cell death induced by STZ, an inducer of apoptotic cell death at low concentrations [41]. STZ is an alkylating agent that damages DNA and generates reactive oxygen species, leading to impairment of proteins involved in glucose metabolism [29], [42], while GKA enhances glucose metabolism by activating glucokinase. These may be partially responsible for the protective effect of piragliatin under STZ treatment even if temporary.

Unlike exenatide, piragliatin showed no anti-apoptotic effects in Tg-induced beta cell death. In our unpublished studies, various GKAs with different pharmacophores exhibited same beta cell protection effects, indicating that this effect is not chemically specific. Several studies suggested that glucokinase might play a role in the regulation of apoptosis as a pro-apototic partner of Bad in liver [43] and beta cells [44]. However, pharmacological challenge with GKA failed to prevent islet apoptosis but increased beta cell mass *in vivo* [23], indicating that the increase of beta cell mass was not a product of anti-apoptotic effect. GKA also promotes the expression of Pd×1 and IRS2, related to beta cell proliferation and survival in mice islets [45], but long-term treatment with GKA failed to preserve beta cell mass [24]. Accumulated evidence and our findings together, suggest that GKA cannot fully compensate for beta cell loss elicited by a variety of physiological stresses.

Additionally, we found that isoform-specific regulation of 14-3-3β, ε, and θ correlates well with beta cell death and that this phenomenon is completely blocked by exenatide treatment. Although activated JNK phosphorylates 14-3-3β, ε, σ, and ζ in Bax-induced apoptosis [46], the phosphorylation sites reported are different from those noted in our studies. Moreover 14-3-3θ was not included, because it does not contain the putative JNK phosphorylation site, ^186^Ser-Pro (designated No. for β) [46]. We could not assess the modifications of 14-3-3 due to the lack of specific antibodies that can detect specific modifications. Most of previous studies mainly proposed the interaction of 14-3-3 with phosphorylated partner proteins [47]. We first identified the specific modifications of 14-3-3β, ε and θ, whereby JNK may not be a major player. We are now examining whether the shifting of the spots with changes in beta cell death is attributable to their modifications. Further studies are needed to determine which kinases mediate the modifications and what molecular interactions are affected by these modifications of 14-3-3 protein.

Protein 14-3-3θ is widely distributed and abundantly present in cells [48]. However, few reports are available so far on its isoform-specific function in general; none on its role in beta cell survival or death. Here, we demonstrated the contribution of 14-3-3θ to beta cell viability, and showed that exenatide treatment preserved the levels of 14-3-3θ under stress conditions. Additionally, INS-1 cells were shown to be more susceptible to stress-induced death at the lower levels of 14-3-3θ. Our findings are in line with a previous study reporting a gradual down-regulation of 14-3-3θ isoform in apoptotic astrocytes [49], which suggests a specific role of 14-3-3θ in cell survival.

**Figure 8:**
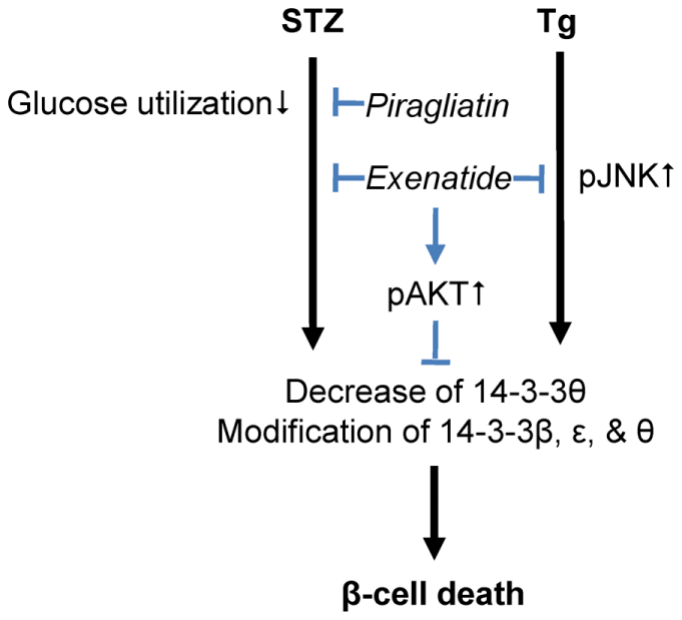
A proposed scheme of protection effects of exenatide and piragliatin in beta cell death.

To sum up as shown in Fig. 8, the present study supports the hypothesis that exenatide and piragliatin exhibit different effects on beta cell death under ER and oxidative stresses respectively by different mechanisms. This study is the first to suggest that JNK-independent 14-3-3 modifications may play a key role in beta cell apoptosis and more importantly, that preservation of 14-3-3θ proteins is needed for beta cell survival.

## Supporting Information

Figure S1 None of exenatide and piragliatin showed cytotoxic effect in INS-1 cells following 24 hr-treatment.
(DOCX)
