# Supplementary material for: Differential Protective Effects of Exenatide, an Agonist of GLP-1 Receptor and Piragliatin, a Glucokinase Activator in Beta Cell Response to Streptozotocin-Induced and Endoplasmic Reticulum Stresses
Source: PLoS One. 2013 Sep 19;8(9):e73340. doi: 10.1371/journal.pone.0073340 (PMC3777936; doi:10.1371/journal.pone.0073340)
Supplement: Figure S1 — None of exenatide and piragliatin showed cytotoxic effect in INS-1 cells following 24 hr-treatment. (DOCX) [file pone.0073340.s001.docx]

**Supplementary Figure 1.** None of exenatide and piragliatin showed cytotoxic effect in INS-1 cells following 24 hr-treatment.

**A B**

After INS-1 cells were treated with exenatide or piragliatin alone in RPMI media containing 5.6 mM glucose, cellular ATP levels and caspase-3/7 activity was assessed in separate experiments as described in Materials and Methods. Exenatide and piragliatin had no effects on cellular viability and did not induced apoptosis up to 0.1 μM and 100 μM, respectively.
